# *Salmonella* Typhimurium's Transthyretin-Like Protein Is a Host-Specific Factor Important in Fecal Survival in Chickens

**DOI:** 10.1371/journal.pone.0046675

**Published:** 2012-12-21

**Authors:** Sarah C. Hennebry, Leanne C. Sait, Raju Mantena, Thomas J. Humphrey, Ji Yang, Timothy Scott, Andreas Kupz, Samantha J. Richardson, Richard A. Strugnell

**Affiliations:** 1 Department of Biochemistry and Molecular Biology, Bio21 Molecular Science and Biotechnology Institute, The University of Melbourne, Victoria, Australia; 2 Department of Clinical Veterinary Science, The University of Bristol, Langford House, Langford, Bristol, England; 3 Department of Microbiology and Immunology, The University of Melbourne, Parkville, Victoria, Australia; 4 School of Medical Sciences, RMIT University, Bundoora, Victoria, Australia; Charité, Campus Benjamin Franklin, Germany

## Abstract

The transthyretin-like protein (TLP) from *Salmonella enterica* subspecies I is a periplasmic protein with high level structural similarity to a protein found in mammals and fish. In humans, the protein homologue, transthyretin, binds and carries retinol and thyroxine, and a series of other, unrelated aromatic compounds. Here we show that the amino acid sequence of the TLP from different species, subspecies and serovars of the *Salmonella* genus is highly conserved and demonstrate that the TLP gene is constitutively expressed in *S.* Typhimurium and that copper and other divalent metal ions severely inhibit enzyme activity of the TLP, a cyclic amidohydrolase that hydrolyses 5-hydroxyisourate (5-HIU). In order to determine the *in vivo* role of the *S.* Typhimurium TLP, we constructed a strain of mouse-virulent *S.* Typhimurium SL1344 bearing a mutation in the TLP gene (SL1344 *ΔyedX*). We assessed the virulence of this strain via oral inoculation of mice and chickens. Whilst SL1344 *ΔyedX* induced a systemic infection in both organisms, the bacterial load detected in the faeces of infected chickens was significantly reduced when compared to the load of *S.* Typhimurium SL1344. These data demonstrate that the TLP gene is required for survival of *S.* Typhimurium in a high uric acid environment such as chicken faeces, and that metabolic traits of Salmonellae in natural and contrived hosts may be fundamentally different. Our data also highlight the importance of using appropriate animal models for the study of bacterial pathogenesis especially where host-specific virulence factors or traits are the subject of the study.

## Introduction

*Salmonella enterica* serovar Typhimurium (*S.* Typhimurium) (and other serovars of *Salmonella enterica* subspecies I) survive in the alimentary tract of adult poultry, often without causing significant systemic disease [1]. The subsequent contamination of chicken carcasses during processing is thought to be a major cause of food poisoning. It is estimated that up to 26% of food-poisoning cases reported in the US are due to consumption of *Salmonella*-contaminated food [2]. Understanding the mechanisms which allow *Salmonella* to survive in such an environment is therefore an important aspect of developing strategies which could reduce human food-borne salmonellosis. Currently, much of the research concerning *Salmonella* pathogenesis is extrapolated from studies in the mouse model of enteric fever. However, the genes required for pathogenesis in a murine system (or indeed any mammalian system), are not necessarily those which are required for colonisation of the alimentary tract of poultry. Salmonellae diverged from a common ancestor with *E. coli* between 120 and 160 million years ago [3]. Throughout its evolution, *Salmonella* has been associated with reptilian and avian hosts. The ability of *S. enterica* to colonise and cause disease in mammals is thus likely a relatively recent event in the evolution of these bacteria. Therefore, when examining the roles of proteins which have been conserved throughout the evolution of *Salmonella*, it is also important to take into account the original or perhaps more “native” environment of the organism in the context of a host-pathogen relationship.

**Figure 1:**
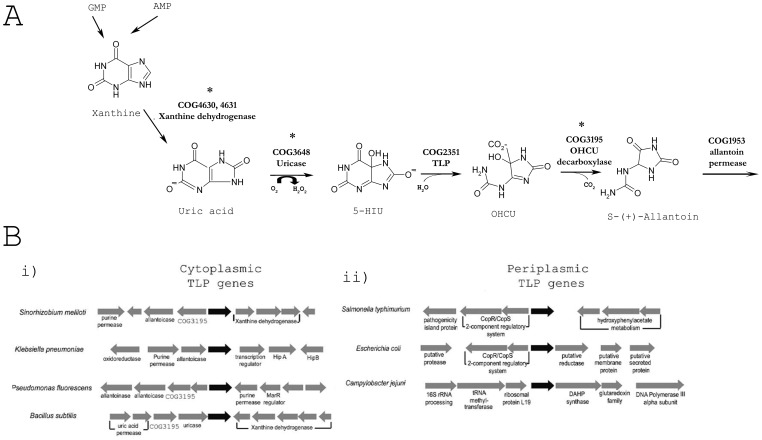
Purine metabolism in bacteria. **A**. Schematic illustrating the enzymes involved in purine metabolism, specifically in conversion of uric acid to allantoin. The COG (cluster of orthologous groups) numbers indicate the protein family which functions at different steps in the pathway. Asterisks denote homologs which have not been identified in the genomes of members of the *Salmonella* genus. **B**. Comparison of the genetic context of i) genes encoding cytoplasmic TLPs with ii) genes encoding periplasmic TLPs. Genes encoding cytoplasmic TLPs are generally located within purine metabolism operons. Genes encoding periplasmic TLPs are not located within operons and do not appear to have a consensus genomic context.

Transthyretin-like protein (TLP; encoded by the gene *yedX*; STM1097) is a hydrolase conserved in all members of the *Salmonella* genus. TLP is an interesting protein insofar that is has been found in all the major kingdoms [4] and is structurally related to the vertebrate thyroid hormone distributor protein, transthyretin [5]. Unlike, transthyretin, TLP functions in the purine catabolism pathway [6]–[9]. Specifically, TLP hydrolyses 5-hydroxyisourate (5-HIU), a metastable compound formed via the enzymatic or non-enzymatic oxidation of uric acid [9], [10]. Upon formation, 5-HIU can spontaneously decompose to the relatively inert compound allantoin, but this process also involves the formation of a variety of radical adducts which have been shown to contribute to lipid peroxidation [11]. When hydrolysed by TLP, 5-HIU is converted to 2-oxo-4-hydroxy-4-carboxy-5-ureidoimadazoline (OHCU) which is then decarboxylated by a member of the COG3195 family to allantoin (see Figure 1 for schematic) [8]. It has been postulated that the role for TLP in this reaction is to rapidly reduce possible oxidative damage which might occur if 5-HIU was allowed to spontaneously decompose under normal conditions [5].

In most bacteria, the TLP gene is located within purine metabolism operons and the gene product is localised to the cytosol (along with other enzymes functioning in this pathway) [5]. Only one study has been published where the TLP gene was mutated to loss of function. In *Bacillus subtilis*, loss of functional TLP results in slow growth of the bacteria in medium containing uric acid [12]. These results are consistent with an important role for TLP in purine metabolism. In Enterobacteria, and more specifically, in *Salmonella*, the TLP is periplasmic [5]. Given that the enzymatic machinery of the purine metabolism pathway is typically localised in the cytosol, we hypothesised that the *Salmonella* TLP may have a different function to the cytosolic TLPs.

We have previously demonstrated that recombinant *Salmonella* TLP can rapidly degrade 5-HIU *in vitro* [6]. In the present study, we examined the *in vivo* role of the Salmonella TLP by constructing a strain of *S.* Typhimurium SL1344 bearing a mutation in the TLP gene (SL1344 *ΔyedX*). We demonstrated that loss of a functional TLP is not required for full virulence of *S.* Typhimurium in mice. However, we demonstrated that TLP is required for the survival of *S.* Typhimurium in chicken faeces, an important step required for transmission of *S.* Typhimurium from host to host. Our findings highlight the need for appropriate models for the study of bacterial pathogenesis.

## Results

### Bioinformatic investigation of purine metabolism genes in Salmonella spp

BLAST results revealed the presence of TLP genes in all *Salmonella* species and serovars sequenced. In all cases, the TLP gene (*yedX*) encodes a predicted periplasmic protein which is 114 amino acids following cleavage of the N-terminal periplasmic localisation sequence. The level of identity between the TLP sequences from different Salmonellae was striking. The TLP sequences from members of *S. enterica* subsp. I are 100% identical with the exception of the TLP from *S.* Typhi, which differs by one residue at position 3 (serine instead of asparagine). The amino acid sequence identity between the TLP from *S. bongori*, a species which diverged from pathogenic Salmonella before the acquisition of SPI-2 (Ochman & Groisman, 1996 IAI 64;5410), and *S.* Typhimurium was 92%. These observations indicate an important functional role for TLP in *Salmonella* and the existence of a strong selection pressure during the evolution of *Salmonella* for retention of a functional TLP.

Interestingly, BLAST searches also revealed that *Salmonella* appear to lack the requisite upstream enzymes in the purine metabolism pathway which convert xanthine to uric acid and uric acid to 5-HIU. Figure 1A illustrates the enzymatic steps required to metabolise xanthine to allantoin and the groups of proteins (COGs) which accomplish this: xanthine dehydrogenase (COG4630, 4631) and uricase (COG3648) (the enzymes acting upstream of the TLP); and OHCU decarboxylase (COG3195) and allantoinase (COG0044) (the enzymes which act downstream of the TLP). The only COG members encoded by the *Salmonella* genome are those from COG2351 (TLP) and COG1953 (*allP*; allantoin permease). These findings suggested that *Salmonella* TLP might have a different function compared to other bacterial (cytosolic) TLPs.

In bacteria, TLP is localised to the cytosol or to the periplasm [5]. In instances where the TLP gene encodes a cytosolic protein, the gene was typically found in a specific genomic context, located in operons encoding the enzymes required for purine metabolism (see Figure 1Bi). In *Salmonella* and related members of the Enterobacteriaceae family, the TLP gene encodes a periplasmic protein. We have previously confirmed the periplasmic localisation of the *S.* Dublin TLP [5]. Periplasmic TLP genes are not found associated with purine metabolism (see Figure 1Bii) or other operons but have been demonstrated to share the same enzymatic function as cytosolic TLP [6], [9].

### The phylogeny of periplasmic TLP genes

**Figure 2:**
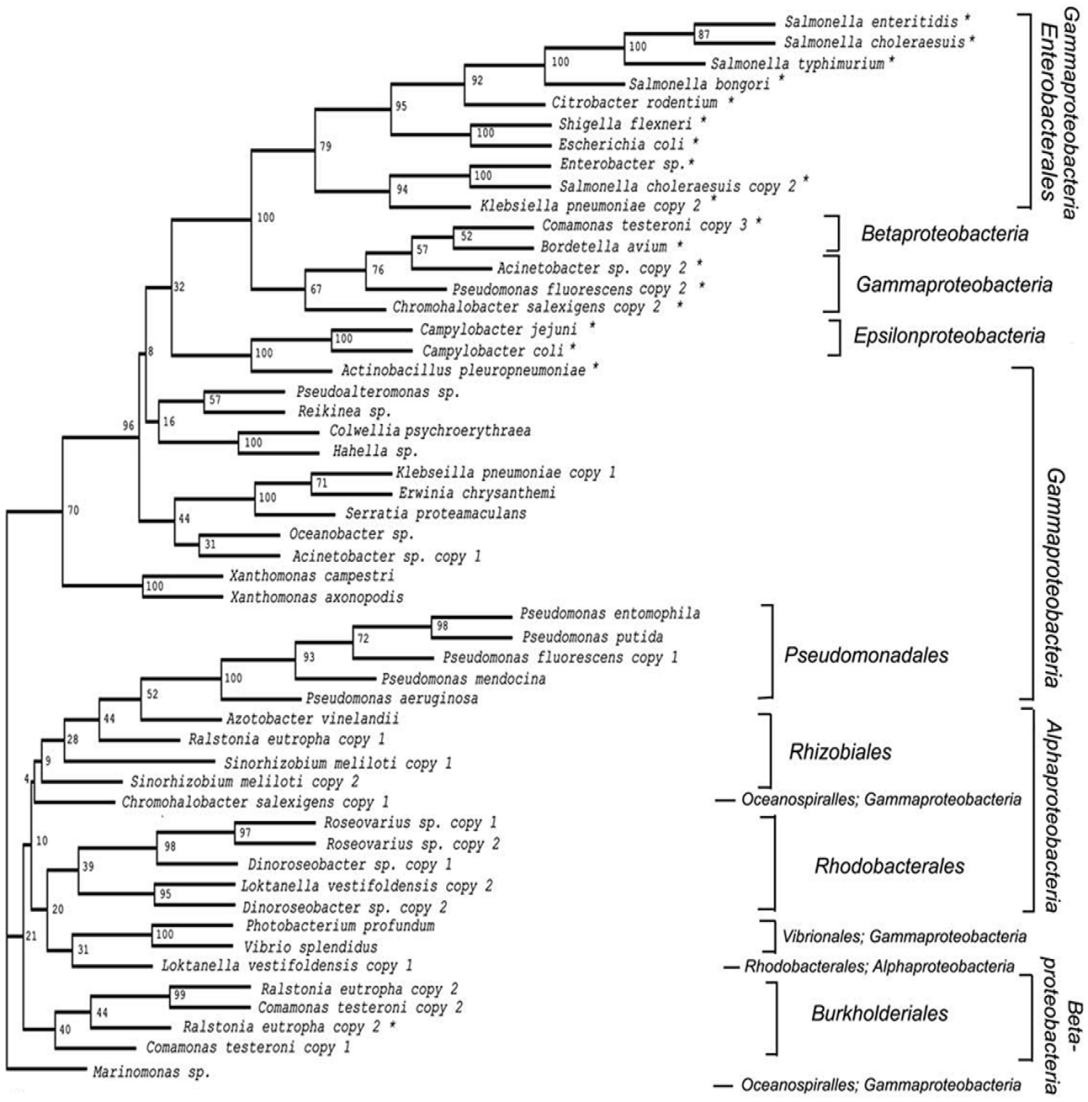
Phylogenetic relationship of cytoplasmic and periplasmic TLPs. Neighbor-joining tree of representative periplasmic and cytosolic TLP sequences from various classes of proteobacteria. One hundred bootstrap replicates were performed to assess the robustness of the tree and the results are indicated at each node in the tree. The TLP sequence from *Marinomonas* sp. was used as the outgroup and to root the tree. Periplasmic TLP sequences are indicated with an asterisk. The taxonomic classes of the bacteria represented in the tree are indicated on the far right hand side of the Figure (Gammaproteobacteria, Betaproteobacteria, Epsilonproteobacteria, Alphaproteobacteria). The taxonomic orders within these subsets are indicated to the immediate right hand side of the tree (Enterobacteriales, Pseudomonadales, Rhizobiales, Oceanospiralles, Rhodobacterales, Vibrionales, Rhodobacterales, Burkholderiales). In the instances where TLP sequences grouped in a region of the tree alongside TLP sequences from unrelated bacteria, both the order and class of the bacterium are indicated (e.g. Oceanospiralles, Gammaproteobacteria). The tree demonstrates that periplasmic TLP sequences evolved along a separate evolutionary pathway to cytoplasmic TLP sequences.

We have previously shown that several bacteria possess more than one copy of the TLP gene, typically one encoding a cytosolic TLP and another encoding a periplasmic TLP [4]. A neighbour-joining tree of cytoplasmic and periplasmic TLPs identified from various proteobacteria (Figure 2) shows that cytoplasmic and periplasmic TLP sequences from the same bacterial species group separately on the tree. The groupings of the periplasmic TLP sequences from distantly related bacteria clearly demonstrate the common relationship between all periplasmic TLP sequences. For example, the TLP sequences from betaproteobacterium *Bordetella avium* and from the epsilonproteobacterium *Campylobacter jejuni*, were found to be closely related to the TLP sequences from members of the *Enterobacteriaceae* family. (We detected only one exception to this observation; in *Ralstonia eutropha*). These data suggest that periplasmic TLP sequences may have originated from an ancestor in the Enterobacteriacae and have since diverged in sequence whilst retaining function in the periplasm.

### Expression and localisation of TLP in *S. Typhimurium*

**Figure 3:**
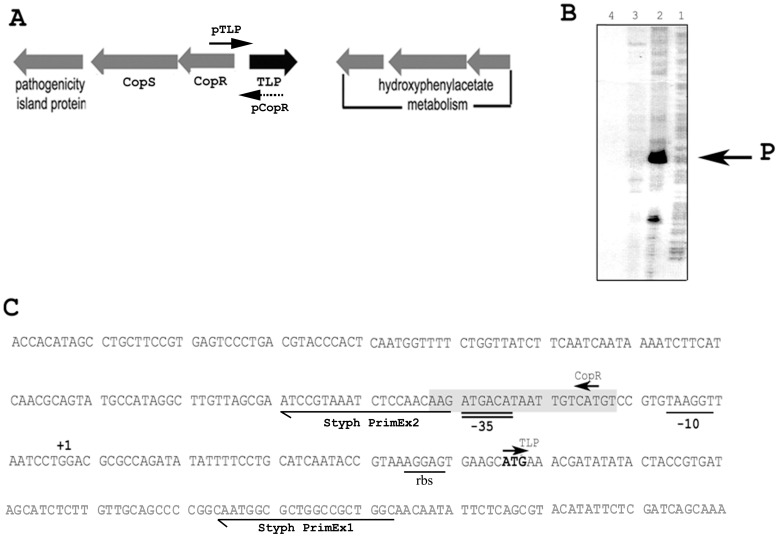
Characterisation of the promoter of the *S.* Typhimurium TLP gene. **A**. The TLP gene promoter overlaps the 5′ untranslated region of the CopR gene. The bold arrow indicates the relative position of the TLP gene promoter and the dashed arrow indicates the relative position of the CopR gene promoter on the complementary strand of DNA. **B**. The results of primer extension analysis using [γ^32^P]-radiolabelled primer StyphPrimEx1 (data not shown) Samples were electrophoresed in an 8% urea/polyacrylamide gel and visualised by exposure of Kodak XAR-5 MR film. In lane 1, the GA ladder is shown; lane 2: reverse transcription product using RNA from strain SL1344/pSCH (see Table 1); lane 3: reverse transcription product using RNA from SL1344; lane 4: no RNA control. Migration of the single product (indicated “P” in lane 2) was compared to the GA ladder and indicated that the start site of transcription was 49 nucleotides upstream of the ATG codon. This was determined by comparing the genomic DNA sequence upstream of the StyphPrimEx1 sequence to the GA ladder. **C**. The DNA sequence upstream and downstream of the ATG (initiation of translation) codon for the *yedX* (TLP) gene is shown. The +1 site (start site of transcription) determined by primer extension analysis is indicated. The −10 and −35 regions of the TLP gene promoter are indicated with a single underline and a double underline, respectively. The shaded grey box represents the putative palindromic copper-box sequence that may influence the transcription of both the TLP and CopR genes. The start site of translation of the CopR gene is indicated (CopR←) and is on the complementary strand to the TLP gene promoter. Thus, the 5′ untranslated region of the CopR gene is also the sequence complementary to the TLP gene promoter.

The transcription of TLP genes located in purine metabolism operons is regulated by purines. For example, in *Deinococcus radiodurans*, the uricase and TLP genes are co-expressed and regulated by a uric-acid dependent MarR-like transcription factor [13]. In order to gain further insight into the function of *S.* Typhimurium TLP, we characterised the promoter of the TLP gene in this organism. Primer extension analysis identified a single strong promoter, 49 nucleotides upstream of the start site of translation (Figure 3). The −10 and −35 regions identified in the promoter of *yedX* were found to share significant sequence similarity with the consensus sequence recognised by σ^70^ transcription factor (Figure 3C). No MarR-like binding site, as found in the TLP gene of *D. radiodurans*, was detected.

Genes induced by nitrogen limitation frequently possess σ^54^-dependent promoters (for a review, see [14]). Consequently, transcription of genes encoding proteins which are part of the same metabolic pathway as TLP e.g. allantoinase and xanthine dehydrogenase, is also regulated by σ^54^ [15]. We did not to detect a σ^54^consensus recognition-site in the TLP gene promoter. This suggested that the principal role of the TLP is not associated with purine salvage in *Salmonella*.

We noted that the promoter of the TLP gene overlapped the promoter of copper-sensitive two-component system encoded on the complementary strand (Figure 3A and 3B). We constructed a P*_TLP_*-lacZ strain of *S.* Typhimurium to test the responsiveness of the TLP promoter to copper present in the growth medium. We could not detect any significant change in the activity of the promoter in M9 minimal media supplemented with up to 500 µM CuCl_2_ (data not shown).

### In vitro function of *S. Typhimurium* TLP

TLP is a cyclic amidohydrolase (E.C. 3.5.2). Most proteins in this family require a divalent metal cation cofactor for activity and are inhibited by other metal cations [16]. Structural analysis of the *Salmonella* TLP demonstrated that it does not require a metal cofactor [6] but that zinc ions, added during crystal formation, were shown to co-localise to the active site of the *E. coli* TLP [17]. We hypothesised that addition of divalent metal ions to the reaction of TLP with 5-HIU would reduce activity of the protein, as both 5-HIU and metal ions would compete for the same sites on the protein.

**Figure 4:**
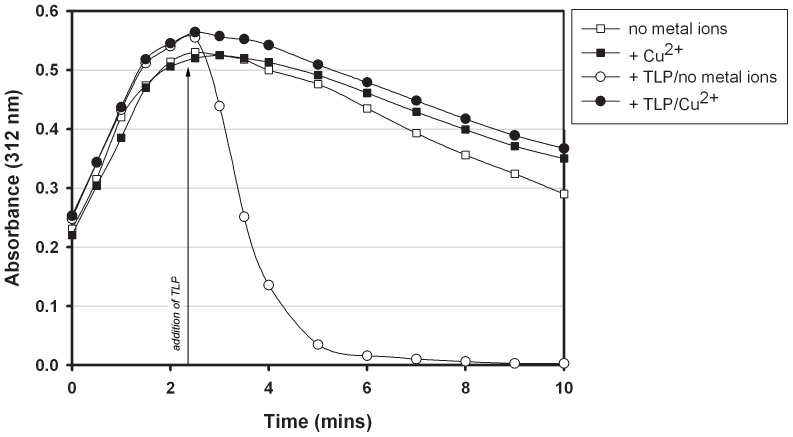
TLP hydrolytic activity in the presence of divalent metal ions. The production and hydrolysis of 5-HIU was measured at 312 nm and is shown over a 10 minute period. Enzyme reactions were performed as described in the text. Briefly, 0.04 U uricase was equilibrated in 50 mM potassium phosphate buffer, pH 7.8 in the presence or absence of 100 µM divalent metal ions (Cu^2+^; results for other divalent metal cations not shown). Enzyme reactions were initiated with the addition of 100 µM freshly diluted uric acid (at time 0). Reactions were performed at 22°C in a Biorad spectrophotometer. Open squares (□): production of 5-HIU in the absence of metal ions. Closed squares (▪): production of 5-HIU in the presence of Cu^2+^. After approximately 3 minutes, the amount of 5-HIU peaked and underwent slow, spontaneous decomposition. Open circles (○): addition of 5.2 nM recombinant *S.* Typhimurium TLP at time 2.5 minutes in the absence of metal ions resulted in rapid hydrolysis of 5-HIU. Closed circles (•): in the presence of Cu^2+^ no hydrolysis occurred following addition of TLP. (Data for other metal ions not shown).

The enzyme kinetics of 5-HIU hydrolysis by TLP were investigated in the presence of various cations. Our data demonstrated that Cu(II) ions are a severe inhibitor of TLP activity (Figure 4). When Cu(II) ions were present at an equimolar concentration to 5-HIU, the activity of the TLP was strongly (approaching 100%) inhibited. Zinc ions were less inhibitory, but still reduced enzyme activity by 92% (data not shown). Copper ions, when present at 2∶1 molar ratio with uric acid, have been reported to be responsible for the oxidation of uric acid [18]. In order to confirm that the copper was not altering the production of 5-HIU, we monitored 5-HIU production following equilibration of uricase with copper. The formation of 5-HIU by the reaction of uric acid and uricase was not affected by the presence of copper. We also tested the effects of Mn^2+^, Ca^2+^ and Mg^2+^ on TLP activity. These metals inhibited TLP activity by 52%, 43% and 14%, respectively (data not shown).

### In vitro characterisation of *S. Typhimurium* TLP mutants

In order to assess if TLP contributed to the survival and virulence of *Salmonella*, a mutation was introduced into the TLP gene (*yedX*) from *S.* Typhimurium SL1344. A comparison of the growth rates of *S.* Typhimurium SL1344 and SL1344 Δ*yedX* strains was performed under a range of *in vitro* culture conditions to determine if there was an obvious phenotype associated with the loss of a functional TLP. These culture conditions included saturating concentrations of uric acid; the results indicated no differences between the strains (data not shown). SL1344 Δ*yedX* exhibited the same responses to oxidative stress (caused by hydrogen peroxide, divalent metal ions, antibiotics, peroxynitrite), temperature changes and reduced nutrient provision as SL1344. The SL1344 Δ*yedX* strain exhibited the same chemotactic and multi-cellular behaviour as SL1344.

**Figure 5:**
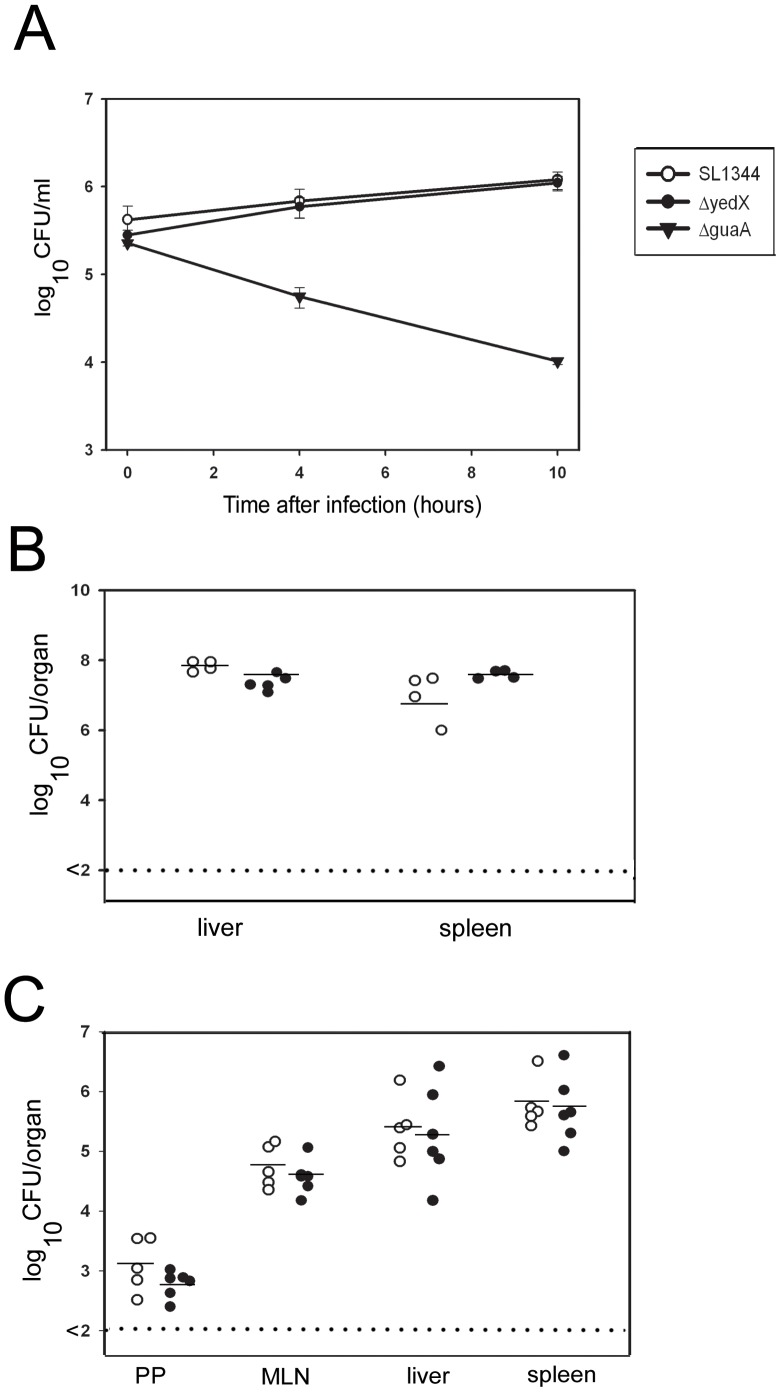
Survival of *S.* Typhimurium strains SL1344 and Δ*yedX* in mouse macrophages and C57BL6 mouse organs. **A**. Survival and growth of *S.* Typhimurium strains SL1344, Δ*yedX* and ΔguaA in RAW 264.7 cells is shown. Open circles (○): number of viable SL1344 bacteria/ml. Closed circles (•): number of viable Δ*yedX* bacteria/ml. Triangles (▾): number of viable Δ*guaA* bacteria/ml. Both SL1344 and Δ*yedX* survived inside the macrophages and proliferated. Δ*guaA* cells were killed (over a period of several hours) after uptake into the macrophages. **B**. The number of viable SL1344 and Δ*yedX* in mouse liver and spleen 5 days following intravenous injection with ∼1×10^7^ cfu of either strain. Open circles (○): number of SL1344 cells per organ (each circle represents the data from a single mouse); closed circles (•): number of Δ*yedX* cells per organ. The horizontal line (-): average number of bacteria per organ. The dotted line indicates the detection limit of the experiment. There was no difference in the numbers of the two strains in liver or spleen of infected mice. **C**. The number of viable bacteria from strains SL1344 and Δ*yedX* in mouse organs following oral inoculation with ∼1×10^7^ cfu of either strain. PP: Peyer's patches. MLN: mesenteric lymph nodes. Open circles (○): number of SL1344 cells per organ; closed circles (•): number of Δ*yedX* per organ. Horizontal line (-): average number of bacteria per organ. The dotted line indicates the limits of detection of the experiment. There was no difference in the numbers of each of the two strains in Peyer's Patches, meseneric lymph nodes, liver or spleen of infected mice.

To determine if TLP facilitated survival of *S.* Typhimurium in a mammalian host, infection studies using a mouse-derived macrophage line (RAW 264.7) were performed using the strains *S.* Typhimurium SL1344, *S.* Typhimurium SL1344 Δ*yedX* and *S.* Typhimurium SL1344Δ*guaA*. The results demonstrate that *S.* Typhimurium SL1344 and SL1344 Δ*yedX* cells were taken up by the macrophages and were able to proliferate (Figure 5A). After 10 hours, the number of viable cells of these bacteria increased from 4.2×10^5^ per well to 1.2×10^6^ per well. There was no difference in the intracellular growth rates of these two strains of bacteria. The negative control, *S.* Typhimurium SL1344Δ*guaA*, a known attenuated strain of *S.* Typhimurium, was killed by the macrophages. The results suggested that TLP is not required for invasion and survival of *S.* Typhimurium within mouse macrophages.

### TLP is not required to induce systemic infection by *S. Typhimurium* in C57BL6 mice

To test whether the TLP gene was required for survival of *S.* Typhimurium in a mammalian host, mice were intravenously injected with the strains *S.* Typhimurium SL1344 and, SL1344 Δ*yedX*. Two groups of five C57BL6 mice (male, 7 weeks of age) were injected in their tail veins with 300 cfu of the appropriate strain of bacteria. Mice were monitored for weight loss and development of enteric fever over five days, then killed. The Liver and spleen was collected from each mouse and the bacterial load in each was determined (Figure 5B). No statistically significant difference in the virulence of the two strains could be detected, as measured by viable count.

C57BL6 mice were also orally infected with 1×10^9^ cfu of either SL1344 or SL1344 Δ*yedX* strains of *S.* Typhimurium. After five days, mice were killed and Peyer's Patches, mesenteric lymph nodes, liver and spleen were collected and the bacterial load in each tissue was determined. There was no difference in the bacterial counts in each organ for mice infected with the strains of *S.* Typhimurium (Figure 5C). Therefore, we concluded that TLP is not essential for the induction of a systemic *S.* Typhimurium infection of mice. If the SL1344 Δ*yedX* had encountered an increased level of oxidative stress, it was not sufficient to reduce the viability of the bacteria. It is therefore likely that Salmonellae have evolved other mechanisms to contend with this increased oxidative stress in mammalian systems, should such stresses inhibit bacterial replication or survival.

### TLP is essential for survival of *S. Typhimurium* in the chicken gastrointestinal tract

Given that the TLP gene has been conserved in the *Salmonella* genus, it is possible that the function of the protein is associated with conditions encountered in non-mammalian species, i.e. acquired before *Salmonella* evolved attributes required for infection of warm-blooded animals. *Salmonella bongori* and *S. enterica* subspecies II-VII colonise the intestines of reptiles, an environment significantly higher in uric acid than the mammalian intestine (due to the fact that reptiles do not metabolise uric acid). Uric acid is found in particularly high levels in the faeces of reptiles and birds (approximately 270 mg/g dry waste [19]). The ability of *Salmonella* and other enteric organisms to survive in the faeces of their host is associated with transmission i.e. their ability to subsequently infect other hosts including which might accidentally ingest the contaminated faeces.

**Figure 6:**
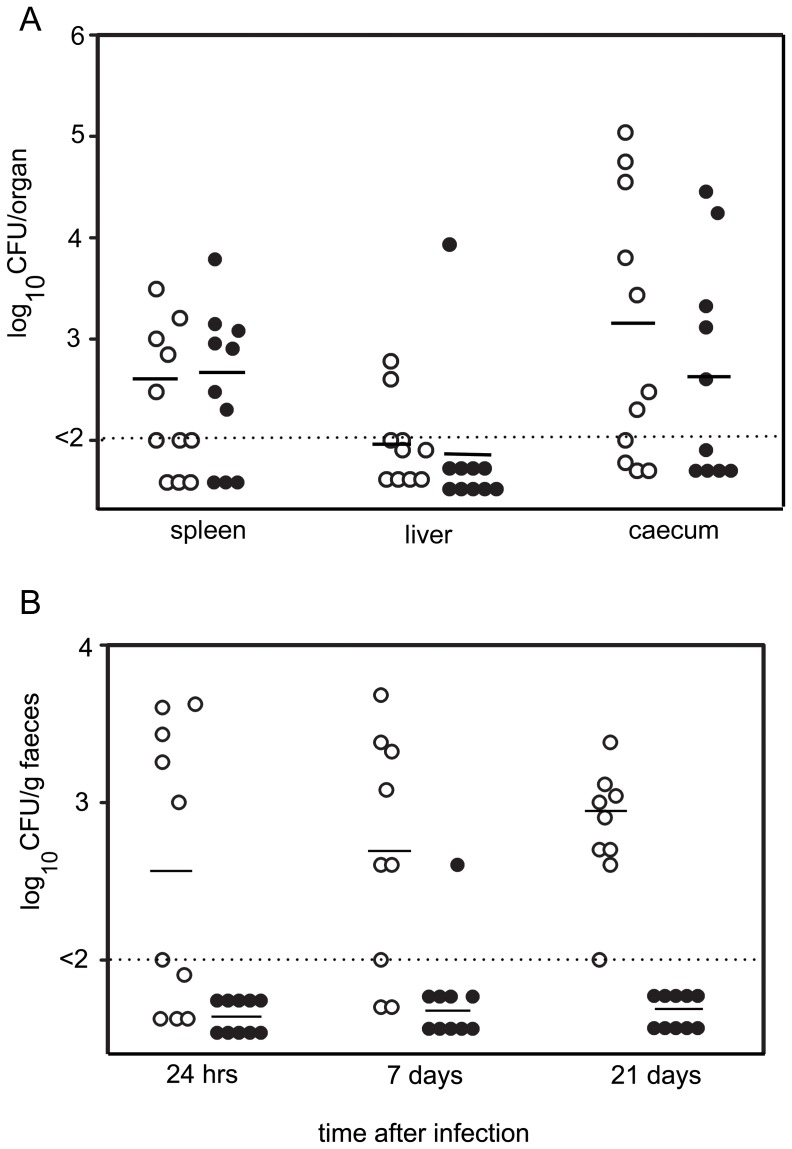
Survival of SL1344 and SL1344 Δ*yedX* in poultry organs and faeces following oral inoculation. **A**. Numbers of viable bacteria in the spleen, liver and caecum of hens following oral inoculation with ∼1×10^8^ cfu of either SL1344 or Δ*yedX* strains of *S.* Typhimurium. Seven days following infection the birds were culled and the bacterial load was determined. Open circles (○): number of SL1344 cells per organ. Closed circles (•): numbers of Δ*yedX* cells per organ. The horizontal line represents the average number of bacteria per organ. The detection limits of the experiment is indicated on the graph with a dotted line. **B**. Numbers of viable bacteria in the faeces of hens up to 21 days following oral inoculation with ∼×10^8^ cfu of either SL1344 or *ΔyedX* strains of *S.* Typhimurium. Open circles (○): number of SL1344 cells per gram faeces. Closed circles (•): numbers of *ΔyedX* cells per gram faeces. The horizontal line represents the average number of bacteria per gram of faeces. The detection limit of the experiment is indicated on the graph with a dotted line. There were significantly lower counts of Δ*yedX* strain in the faeces at all time points compared to SL1344 (Wilcoxon's test, W = 0, p = 0.004).

Hens that are used for commercial production of eggs (16 weeks of age, shaver hens) were orally infected with 1×10^8^ cfu of *S.* Typhimurium SL1344 or SL1344 Δ*yedX*. Seven days following infection the birds were culled and liver, spleen and ceaca were collected for determination of bacterial load. There was no significant difference between the numbers of the two strains of *S.* Typhimurium in these organs (Figure 6A). A second group of birds were infected with the two strains (1×10^8^ cfu) and monitored over a period of 21 days in order to determine the level of faecal shedding. Initially, counts of SL1344 were around 9×10^2^/g faeces and this increased slowly over the period of 3 weeks. There were significantly reduced counts of the *ΔyedX* strain in the faeces of chicken (Wilcoxon's test: p = 0.004) (Figure 6B). These data clearly demonstrate that TLP is required for survival of *S.* Typhimurium in chicken faeces. Furthermore, we demonstrate the importance of choosing appropriate animal models for investigating bacterial survival and virulence.

## Discussion

TLP is one of a relatively small number of proteins that are structurally conserved across the four kingdoms. We have previously demonstrated that a number of residues are 100% conserved throughout TLP evolution and are essential to its enzymatic activity [6]. TLP shares a high level of structural and sequence homology with the vertebrate protein transthyretin [6], which is responsible for the distribution of thyroid hormones (for a review see, [20]). Identification of TLP in bacterial and plant genomes suggested that the original function of the ancestral TLP may lie in central or secondary metabolism. TLP is now known to play an important role in the rapid conversion of oxidised uric acid to allantoin (for reviews, see [4], [21]).

The TLP gene has been identified in all members of the *Salmonella* genus. The degree of divergence of the TLP sequences in *Salmonella* is minimal, with 92% sequence identity between the *S. bongori* and *S.* Typhimurium TLPs. These observations indicate an important functional role for TLP in *Salmonella* and the existence of a strong selection pressure during the evolution of *Salmonella* for retention of a functional TLP.

Salmonellae, unlike other members of the Enterobacteriaceae family e.g. *Klebsiella* spp. and *Enterobacter* spp., lack the genes encoding xanthine dehydrogenase, uricase and OHCU decarboxylase and cannot utilise uric acid as a sole source of nitrogen and carbon [22]. The presence in *Salmonella spp.* genomes of a TLP gene (*yedX*) that can hydrolyse 5-HIU *in vitro* [6] led us to hypothesise that TLP may have a role degrading 5-HIU obtained from the external environment [5].

The end-point of purine metabolism in birds, reptiles, some insects and humans is uric acid. In most other organisms, uric acid is further oxidised to allantoin by three enzymatic steps (see Figure 1A). As a result of their inability to enzymatically oxidise uric acid, birds have high levels of uric acid in the plasma and tissues, which in turn provide several benefits to these animals [23]. Firstly, because uric acid is highly insoluble, birds can excrete it with a minimal amount of water loss. Secondly, uric acid is a potent antioxidant, chelating transition metal ions and quenching free radicals such as peroxynitrite and hypochlorous acid in the plasma and tissues (for review, see [24]). The faecal matter of reptiles and birds therefore contains high levels of uric acid (27% w/w [19]).

*Salmonella* evolved over many millennia to survive in a reptilian gut and subsequently in an avian gut. The ability to colonise the gastrointestinal tract of mammals is a relatively recent event in the evolution of these bacteria (for a review, see [25]). As commensurate organisms of the reptilian and avian gastrointestinal tract, *Salmonella* would have to survive in environments rich in uric acid and its (potentially toxic) oxidation products generated by members of the gut microbiota metabolising urate. In particular, highest counts of *Salmonella* are found in the caecum and cloaca of poultry [26] which are also the regions of the gut highest in uric acid [27]. It would therefore not be surprising if *Salmonella* had evolved a mechanism to detoxify oxidation products of uric acid to which they may be exposed in the caeca and faeces of reptiles and birds.

Our data indicate that TLP is not required for invasion of and proliferation in mouse-derived macrophages; nor for induction of systemic infection in C57BL6 mice. Similarly, TLP was not required for colonisation of the alimentary tract of chickens. We have demonstrated that the activity of recombinant TLP is severely inhibited by divalent cations but cannot show an in vitro phenotype for the *S.* Typhimurium *yedX* mutant. Furthermore, we have demonstrated that loss of a functional TLP results in marked attenuation of *S.* Typhimurium SL1344 survival in the faeces of infected poultry. In *Salmonella*, rather than being required for endogenous synthesis of allantoin, as is the case for bacteria possessing cytoplasmic TLP, periplasmic TLP appears to be required for survival of the bacterium in a high uric acid environment, ie. poultry faeces. We subsequently attempted to show *yedX*-dependent growth differences *in vitro* under a wide variety growth conditions where high uric acid concentrations were present (data not shown). These included Luria broth containing saturating concentration of uric acid (approximately 6 µM), minimal media with saturating uric acid, human urine (as a biological source of uric acid), with and without added glucose, a saturated filtered solution of fresh chicken feces, and Luria broth at pH ranging from 6 to 10, with and without copper sulphate (concentrations ranging from 0.25–2 mM). The *yedX* mutant of SL1344 showed similar growth dynamics to the wild type under each of these conditions. The absence of an *in vitro* growth defect in the *yedX* mutant suggests that it may be necessary to include a fecal microbiota to demonstrate the survival function of *yedX*, a hypothesis that will be difficult to test ex vivo.

We consequently propose a mechanism whereby oxidised uric acid, which is the product of other anaerobic bacteria present in chicken feces, produces 5-HIU that permeates the outer membrane of the bacterium. To protect the cell from lipid oxidation due to the slow decomposition of 5-HIU and its reaction with free radicals, periplasmic TLP rapidly hydrolyses 5-HIU. The allantoin generated in the periplasm can then be imported to the cytoplasm via the inner membrane *allP*-encoded allantoin permease.

## Conclusion

Currently, *Salmonella*-contaminated food supplies represents a major cause of food-poisoning around the world. *Salmonella* spreads among populations of chickens by shedding and ingestion of *Salmonella*-contaminated faeces. We have identified a protein, TLP, which is absolutely required for survival of *Salmonella* in poultry faeces. Our demonstration of the severe inhibition of TLP activity by divalent cations may facilitate the development of novel strategies aimed at reducing *Salmonella* contamination of human food supplies. Our data also highlight deficiencies in the use of the mouse model of *Salmonella* pathogenesis and demonstrate the need for appropriate animal models for the investigation of bacterial pathogenesis since the TLP-related phenotype that was obvious from studies in chickens was not seen in the murine model.

## Experimental Procedures

### Bioinformatic and Phylogenetic Analyses

BLAST searches were performed against Comprehensive Microbial (http://cmr.tigr.org), NCBI nr (http://www.ncbi.nlm.nih.gov/blast) and Sanger (http://www.sanger.ac.uk) databases using the previously published *S. dublin* TLP sequence [5] as the query sequence. Phylogenetic analyses were performed as previously described [5].

### Bacterial strains, media and growth conditions

The bacterial strains and plasmids used in this study are listed in Table 1. Bacteria were grown in LB Broth or on LB plates at 37°C under aerobic conditions throughout the study, unless otherwise stated. Antibiotics were used as needed at the following concentrations: ampicillin, 100 µg ml^−1^; kanamycin, 50 µg ml^−1^; streptomycin, 50 µg ml^−1^; trimethoprim, 40 µg ml^−1^. Cell growth was monitored by measuring the turbidity at 600 nm. Where appropriate, M9 minimal media [28] supplemented with 0.02% glucose was utilised (using C5 *S.* Typhimurium derived strains only).

**Table 1:** Plasmids and bacterial strains used.

Plasmid	Characteristics	Source or Reference
pKD46	expression vector for phage λ Red recombinase	[7]
pMU2385	low copy plasmid with promoter-less lacZ gene	[11]
pMU2385-*pTLP*	pMU2385 carrying *yedX* promoter	This work
pMU2385-*pCopR*	pMU2385 carrying CopR gene promoter	This work
pUC4-Kiix	Vector containing Kanamycin resistance gene	Pharmacia
pAT153	Mid-copy plasmid stable in *Salmonella* Typhimurium	[9]
pBSK+	cloning vector	Stratagene
pSCH1	pBSK containing *S.* Typhimurium *yedX*	This work
pSCH2	pSCH1 with KanamycinR cassette inserted in *yedX*	This work
pAT153-*yedX*	plasmid for complementation of SL1344 *yedX*::Kan^R^	This work
pET11a	expression vector	Novagen
pET11a-TLPm	pET11a carrying the sequence of *S.* Dublin TLP gene (*yedX*)	[16]
P22 HT105/1-int	non-lysogenic, transducing bacteriophage	Salmonella Stock Centre

Strain	Characteristics
LB5010	*S.* Typhimurium; rough LPS, restriction^−^ and modification^+^
SL1344	*S.* Typhimurium wild-type
SL1344 pSCH1	SL1344 carrying pSCH1 plasmid; for *yedX* RNA for primer extension
SL1344 pAT153	Wild-type carrying control pAT153 plasmid
SL1344 *ΔyedX*	SL1344 *yedX* :: Kan^R^
SL11344*ΔyedX* pAT153	Δ*yedX* carrying pat153; control for pAT153 plasmid
SL1344 *ΔyedX* c’	complemented mutant; Δ*yedX* carrying pAT153 *yedX*
P*TLP*-lacZ	SL1344 transformed with pMU2385 - P*TLP lac Z* construct
P*CopR*-lacZ	SL1344 transformed with pMU2385 - P*CopR lacZ* construct
SL1344 pMU2385	SL1344 transformed with pMU2385 (promoterless *lacZ*)

### Generation of ΔyedX SL1344

The *ΔyedX* strain of SL1344 was generated according to the method of [29]. Firstly, the kanamycin resistance cassette from the vector pUC4-Kixx (Pharmacia, GE Healthcare, Chalfont St. Giles, UK) was introduced into the TLP gene sequence of *S.* Typhimurium LB5010 (a restriction^−^ modification^+^ strain). The mutation was then P22 phage transduced onto an SL1344 or C5 background [30]. The success of the mutation and transduction were confirmed by PCR with primers flanking and nested within the TLP gene (data not shown).

### TLP promoter activity

The P*_TLP_*-lacZ strain was generated by ligating a 320 nucleotide fragment of SL1344 genomic DNA containing the TLP promoter into the promoter-less lacZ vector, pMU2385 [31]. This construct was sequenced and then transformed into *E. coli* DH5α to confirm the successful generation of the construct. For TLP promoter activity assays, the construct was transformed into either SL1344 or C5 strains of *S.* Typhimurium. The promoter region was defined as the reverse complement of the TLP gene promoter.

β-galactosidase activity was determined according to the method of Miller [32]. For these experiments, bacterial strains were grown to various cell densities in either LB broth or M9 minimal medium. For growth in M9 media, the C5 strain of *S.* Typhimurium was utilised so that histidine was not required in the medium.

### Primer extension

Primer extension of the TLP mRNA was performed using the method of Hudson and Davidson [33]. To generate sufficient quantities of template, the region of SL1344 DNA comprising the TLP gene and 500 bp flanking sequence (on either side) was ligated into the high copy vector, pBSK (Stratagene, La Jolla, CA, USA). This construct was then transformed into SL1344. This strain was grown in LB broth at 37°C to mid-log phase before RNA was isolated using a Qiagen RNAeasy kit (Qiagen, Germantown, MD, USA).

### TLP activity assays

Recombinant TLP was synthesised and purified as previously described [6]. TLP enzyme activity assays were performed in 1 ml quartz cuvettes at 22°C. Uricase (from *Candida sp.*, at 0.04 U/ml; Sigma-Aldrich, St. Louis, MO, USA) was briefly equilibrated in 50 mM potassium phosphate buffer, pH 7.8. The generation of 5-HIU was initiated with the addition of 100 µM freshly diluted uric acid (MP Biomedicals, Solon, OH, USA) and monitored at 312 nm. For metal inhibition studies, 100 µM of either CuCl_2_, ZnOAc_2_, MnCl_2_, CaCl_2_, MgCl_2_ was included in the equilibration buffer prior to addition of uric acid. TLP (5.2 nM) was added to the reaction mixture at the 2.5 minute time-point to hydrolyse 5-HIU.

### Initial phenotypic characterisation of ΔyedX SL1344

To compare the *in vitro* growth characteristics of wild-type *S.* Typhimurium (SL1344) and the TLP-deficient strain *ΔyedX SL1344*, the two strains were firstly grown in LB medium, with shaking at 37°C. At various time points, aliquots were removed from the cell cultures to determine and compare culture turbidity (OD 600 nm) and the number of colony forming units (cfu) through plating of dilutions onto LB/streptomycin or LB/kanamycin plates. This was repeated under a variety of different conditions: altering temperature (to 30°C or 42°C), altering the growth medium (to M9 minimal salts), altering the pH of the medium (to pH 4, 7 and 9), addition of hydrogen peroxide (at various concentrations up to 15 mM), addition of copper chloride (up to 10 mM), addition of uric acid (up to 1 mM). To compare the motility of the two strains of bacteria, swarming tests were performed in LB medium supplemented with 0.02% glucose and containing 0.6% (w/v) agar. One µl of the appropriate strain (from an overnight culture) was aliquoted into the centre of a plate. The plates were incubated for 16–14 hours at 37°C. Swarming was monitored by visually examining the diffusion of the bacteria away from the initial site of aliquot.

### Characterisation of the growth of ΔyedX SL1344 in mouse- derived macrophages

The cell culture conditions for maintaining the RAW 264.7 macrophage-like cell line have been described previously [34]. Briefly, one day before infection, 118 cells were harvested, counted with a Coulter ZTM Series cell counter (Beckman Coulter, Fullerton, CA) and seeded at 5×105 cells/well in 24-well tissue culture plates. After the cells adhered to the bottom of the wells, they were treated with 2 ml/well of recombinant interferon- (100 units/ml) for 21–24 h. Cultures of SL1344, *ΔyedX SL1344* and SL1344ΔguaA were prepared from frozen stocks in LB and grown for 18 h at 37°C, with shaking. (The *S.* Typhimurium SL1344ΔguaA strain is an attenuated strain, i.e. unable to invade macrophages and cause illness in mice. See Table 1). Bacteria were harvested, washed once with the same volume of Dulbecco's phosphate-buffered-saline (DPBS), and resuspended in 1 ml of DPBS. After the cell density was determined, bacterial cells were diluted in Dulbecco's modified Eagle's medium (DMEM) supplemented with 10% heat-inactivated fetal bovine serum and incubated on ice for 30 min. After macrophage cells were washed twice with 2 ml/well of Hanks' buffered saline solution (HBSS), 1 ml of the bacteria in DMEM was added in each well with a multiplicity of infection of 100. Uptake of bacteria by the macrophages was allowed to occur for 30 min at 37°C in 5% CO2. This time point was defined as 0 h post-infection (p.i.). After cells were washed three times with 2 ml/well of HBSS to remove the bacteria that were not taken up by the cells, 2 ml of DMEM supplemented with fetal bovine serum and 12.5 µg/ml of gentamycin was added to each well. At 0, 4 and 10 hours post-infection, macrophage cells were washed twice with 1 ml/well of HBSS and lysed with 0.5 ml/well of cell lysis buffer (1% (v/v) of Triton X-100, 0.1% 119 (w/v) SDS in DPBS) at room temperature for 5 min. The lysis solution in each well was pipetted up and down 10 times and diluted by several orders of magnitude, and each dilution was plated on LB agar plates supplemented with streptomycin (50 µg/ml) or kanamycin (50 µg/ml). After incubation at 37°C for 16 h, the numbers of colony-forming units on each LB agar plate were counted to determine the numbers of live *S.*

Typhimurium cells in each well, and a minimum of three wells was counted for each measurement.

### Animal infections

*S.* Typhimurium SL1344 and SL1344 *ΔyedX* were grown to mid-log phase in LB liquid medium at 37°C with shaking (180 o.p.m.). The bacteria were then diluted in PBS (phosphate buffered saline) to an approximate concentration of 30 cfu/µl. Five male C57BL6 mice at seven weeks of age were intravenously injected (into the tail vein) with 100 µl of either of the strains. The exact inoculum was determined by plating out dilutions of the bacteria onto LB/agar plates and counting the number of colonies formed following overnight incubation of the plate at 37°C. Five days post-infection, the mice were euthanized with CO_2_ and liver and spleen were harvested. Organs were homogenised in 5 ml PBS at room temperature using a stomacher (Tekmar, Cincinnati, OH, USA) and serial dilutions were plated onto LB/ampicillin or LB/streptomycin plates. Following overnight incubation of the plates at 37°C, the number of bacteria per plate were determined, multiplied by the appropriate dilution factor and used to estimate the number of bacteria per organ.

Bacterial cultures for oral inoculation of mice were grown in 10 ml LB broth at 37°C without shaking for 24 hours. The cultures were then diluted 1/20 (to approximately 1×10^7^ cfu/100 µl) in PBS. Male C57BL6 mice (Jackson laboratory, USA) at 6 weeks of age were used. Prior to infection, mice were given an oral dose of 100 µl of 10% (w/v) sodium bicarbonate. The mice then received 1×107 cfu of either SL1344 or *ΔyedX* strains (as determined by serial dilution of the inocula) by oral gavage. Mice were monitored daily and culled after 5 days. Mesenteric lymph nodes, Peyer's Patches, liver and spleen were collected from each mouse. Peyer's Patches were incubated in 1 ml of 100 µg/ml gentamycin for 1 hr at 37°C. Bacterial counts for all tissue samples were determined as described above.

For infection of chickens, 16 week-old shaver hens were used (a commercial egg layer-type hen). Birds were housed in groups of ten. Infection was by oral gavage (no bicarbonate was given prior to gavage). Birds were infected with either SL1344, *ΔyedX* or *ΔyedX* complemented strains with a dose of approximately 8×10^8^ cfu (as determined by serial dilution and plating of the inocula). Twenty birds were infected with each strain. One week after infection, half of the birds (10 from each group) were culled and liver, spleen and caeca were collected for enumeration of bacteria. The second group of birds was retained for three weeks for enumeration of bacteria in the faeces.

Faecal samples were collected by placing hens in cages and collecting faeces over 21 days. Bacteria were enumerated by weighing the faeces and then dissolving it in buffered peptone water (Oxoid, Hampshire, UK) (9 ml per gram of faeces). Serial dilutions of the dissolved faeces were made and 100 µl of each different dilution was plated onto modified brilliant green agar (Oxoid, Hampshire, UK) containing the appropriate antibiotic. Plates were incubated overnight at 37°C. The number of pink colonies on the plates was counted the following day and used to calculate the number of cfu/g faeces.

### Statistics

For experiments involving chickens, the Wilcoxon signed rank test was performed [35].
